# High Sensitivity pH Sensor Based on Porous Silicon (PSi) Extended Gate Field-Effect Transistor

**DOI:** 10.3390/s16060839

**Published:** 2016-06-07

**Authors:** Naif H. Al-Hardan, Muhammad Azmi Abdul Hamid, Naser M. Ahmed, Azman Jalar, Roslinda Shamsudin, Norinsan Kamil Othman, Lim Kar Keng, Weesiong Chiu, Hamzah N. Al-Rawi

**Affiliations:** 1 School of Applied Physics, Faculty of Science and Technology, Universiti Kebangsaan Malaysia (UKM), 43600 Bangi, Selangor, Malaysia; linda@ukm.edu.my (R.S.); insan@ukm.edu.my (N.K.O.); karkeng.iamkklim@gmail.com (L.K.K.); 2 School of Physics, Universiti Sains Malaysia (USM), 11800 Penang, Pulau Pinang, Malaysia; naser@usm.my; 3 Institute of Microengineering and Nanoelectronics (IMEN), Universiti Kebangsaan Malaysia (UKM), 43600 Bangi, Selangor, Malaysia; azmn@ukm.edu.my; 4 Low Dimensional Materials Research Centre, Department of Physics, Faculty of Science, University of Malaya, 50603 Kuala Lumpur, Malaysia; w.s.chiu@um.edu.my; 5 School of Electrical and Electronic Engineering—Universiti Sains Malaysia (USM), 14300 Nibong Tebal, Pulau Pinang, Malaysia; comeon500@yahoo.com

**Keywords:** macroporous materials, ionic conductivity, pH sensitivity

## Abstract

In this study, porous silicon (PSi) was prepared and tested as an extended gate field-effect transistor (EGFET) for pH sensing. The prepared PSi has pore sizes in the range of 500 to 750 nm with a depth of approximately 42 µm. The results of testing PSi for hydrogen ion sensing in different pH buffer solutions reveal that the PSi has a sensitivity value of 66 mV/pH that is considered a super Nernstian value. The sensor considers stability to be in the pH range of 2 to 12. The hysteresis values of the prepared PSi sensor were approximately 8.2 and 10.5 mV in the low and high pH loop, respectively. The result of this study reveals a promising application of PSi in the field for detecting hydrogen ions in different solutions.

## 1. Introduction

Porous silicon (PSi) is a promising candidate for several applications due to the ease of fabrication and controllable pore size. Furthermore, it is compatible with conventional silicon processing technology [1]. Moreover, PSi attracted the attention of several groups in the past years for several applications. The surface modification of the silicon wafer plays a major role in the sensitivity enhancement of silicon (Si) toward several analytes, and it was successfully deployed in the development of a large variety of biosensors [2,3,4,5,6]. Furthermore, several applications of PSi were investigated, such as light emitted diodes (LED) [7,8,9], photodetectors [10,11,12], waveguides [13], and chemical sensors for detection of various chemicals [5,14,15,16,17]. Furthermore, PSi exhibits a number of properties that make it an attractive material for controlled drug delivery applications [18]. The H^+^ ions (pH) are crucial parameters in biomedical research. A few methods were conducted to measure the pH of the solutions; among them is the extended gate field-effect transistor (EGFET). With this method, a commercial metal–oxide–semiconductor field-effect transistor (MOSFET) is isolated from the chemical environment, and a chemical sensing membrane is located in the media and connected to the gate of the MOSFET. With this configuration, the MOSFET can be used many times without any damage as compared to the ion sensitive field-effect transistors (ISFETs), where the metallic gate of the MOSFET is replaced by a sensitive membrane [19,20,21]. It is worth noting that the theoretical framework of both platforms is essentially the same [22,23].

Several materials were tested as a sensing membrane, among them the metal oxide semiconductors such as zinc oxide [24,25], titanium dioxide [26], palladium oxide [23], and ruthenium dioxide [27]. However, some metal oxides demonstrate poor performance and need special treatments to be used as membranes for extreme pH values [22,24,28,29]. The cost effective preparation method, ease of fabrication of the PSi compared with the metal oxide semiconductors, and the rigid property of the silicon wafer (as a substrate) make it preferable in biosensing applications.

Porous silicon has previously been employed for pH sensing in different platforms. Zehfroosh *et al.* [21] prepared ISFET with PSi on the gate region. The calculated pH sensitivity was 300 mV/pH in the pH range of 4 to 9. This value of the sensitivity was higher than the theoretical value of the Nernst limit (59 mV/pH). Furthermore, it was shown that increasing the drain current enhanced the sensitivity values. The PSi-based potentiometric method was employed for an enzymatic biosensor by Rebby *et al.* [30,31]. The PSi was tested under different pH environments. The sensitivity was calculated to be 30 mV/pH in the pH range of 4.6 to 8.09. A capacitive pH sensor based on PSi was prepared by Schöning *et al.* [32]. The pH sensitivity was found to be 60 mV/pH in the range of pH = 4 to 9 with a hysteresis value of 3 mV.

The sensitivity of the sensing membrane depends on the change of the surface potential voltage (Ψ) between the sensing layer and the electrolyte interface. This is based on the site binding theory [33]. In this theory, the number of binding sites residing on the sensing membrane could lead to changes in the surface potential voltage between the sensing layer and the electrolyte interface. This can be expressed as [29,34,35]:

(1) $$\Psi =2.303\frac{kT}{q}\;\frac{\beta }{\beta +1}(pH_{pzc}-pH)\;$$

where pH_pzc_ is the pH value at the point of zero charge, *q* is the electron charge, *k* is the Boltzmann constant, *T* is the absolute temperature, and β is the sensitivity parameter, which can be calculated using [36];

(2) $$\beta =\frac{2q^{2}N_{s}\sqrt{\left(\frac{K_{a}}{K_{b}}\right)}}{kTC_{DL}}$$

where N_s_ represents the surface site density and C_DL_ is the capacitance of the electrical double layer, as derived from the Gouy–Chapman–Stern model [35]. By increasing the sensitivity parameter β, a better linear response between the surface potential voltage and pH value can be obtained [36].

The theoretical limit value of the pH voltage sensitivity is known as the Nernst limit, and it is equal to 59 mV/pH [22,23,26]. However, in order to solve this problem, several published works proposed different approaches to enhance the voltage sensitivity and cross the Nernst limitation. The enhancement of the voltage sensitivity was proposed via increasing the sensing gate surface area in the ISFET configuration, where a remarkable increase in the measured pH sensitivity was noticed, and it reached 130 mV/pH [37]. The porous nature of the sensing membrane proves to be another approach in enhancing the pH sensitivity. The porous structure of the membrane will increase the accumulation of positive charges on the gate region. The pH sensitivity achieved a value of 300 mV/pH for ISFET platform [21]. Furthermore, a dual-gate ISFET platform was proposed by Spijkman *et al.* [38]. The pH voltage sensitivity was reported to be 2.25 V/pH. With this type of configuration, a capacitive coupling effect is induced between the top and bottom gate capacitance that will result in the enhancement in pH sensitivity [34,38].

According to Yao *et al.* [22], the variation of the surface charge density with interfacial potential for the membrane surface is probably the reason for the departure from the Nernst equation. The porous nature of the membrane surface will increase the charge accumulation and play a major role in enhancing the capability of the membrane in sensing the hydrogen ions.

In this report, we investigate PSi as a membrane for the EGFET pH measurement applications. To the best of our knowledge, the PSi pH sensor as an EGFET platform has not been previously investigated. The performance in the sensitivity and stability of PSi in different buffer solutions of pH will be explored.

## 2. Materials and Methods

### 2.1. The Porous Silicon Formation

**Figure 1:**
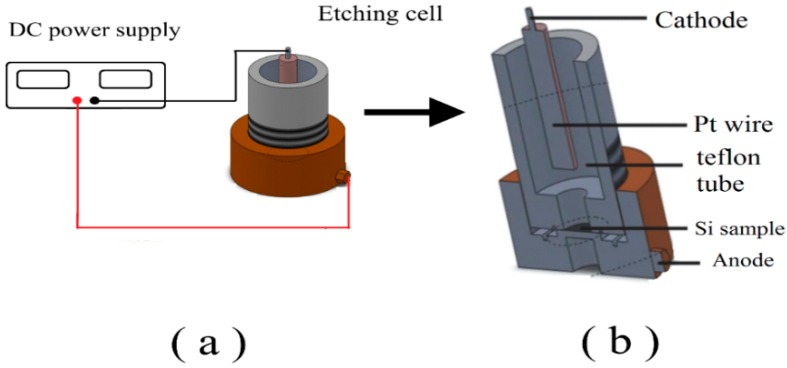
Experimental setup for PSi (**a**) and cross section of the etching cell (**b**).

The PSi layer was prepared via anodizing an n-type silicon (Si) wafer with orientation (100) and resistivity of 0.75–1.25 Ω·cm. The Si wafer was cleaned according to the standard procedure following the RCA method. The wafer was then cut into four pieces and inserted as an anode in the electrochemical etching cell. The polished side was facing the cathode (platinum wire with a diameter of 0.25 mm). The DC photo-electrochemical etching bath was conducted at room temperature in a Teflon^®^ cell containing a mixture of hydrofluoric acid (HF 48%) and ethanol (C_2_H_5_OH 98%) with a volume ratio of 1:4. The PSi layer was formed with a DC constant current at a current density of 20 mA/cm^2^ for 15 min under illumination from a 100 W tungsten lamp; the lamp was fixed at a distance of 20 cm. The samples were then rinsed with deionized water and dried with nitrogen gas. Figure 1 shows a schematic diagram of the anodization system used for the formation of PSi [39].

### 2.2. The Sensor Chip Fabrication

**Figure 2:**
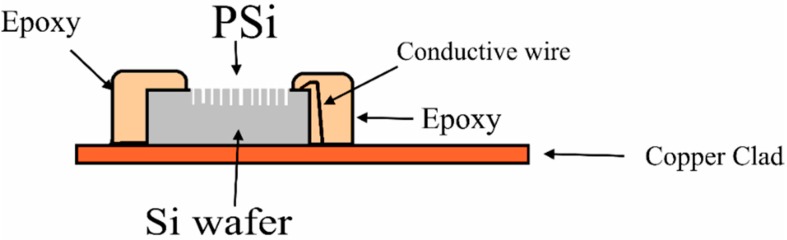
The fabricated PSi sensor on a copper-clad sheet.

Samples of 1 cm × 1 cm in size were scribed from the Si wafer with PSi. The samples were mounted to a strip of a copper clad laminate printed circuit board (PCB) [23]. Conductive wire was connected between the Si wafer and the copper plate using silver paste. Epoxy resin was employed to encapsulate the sensor chip and the copper line of the clad sheet PCB to reduce the leakage current. An area of 0.3 cm × 0.3 cm was free from epoxy and was used as a sensing window. Figure 2 presents the PSi membrane after encapsulation.

### 2.3. Measurement Processes

**Figure 3:**
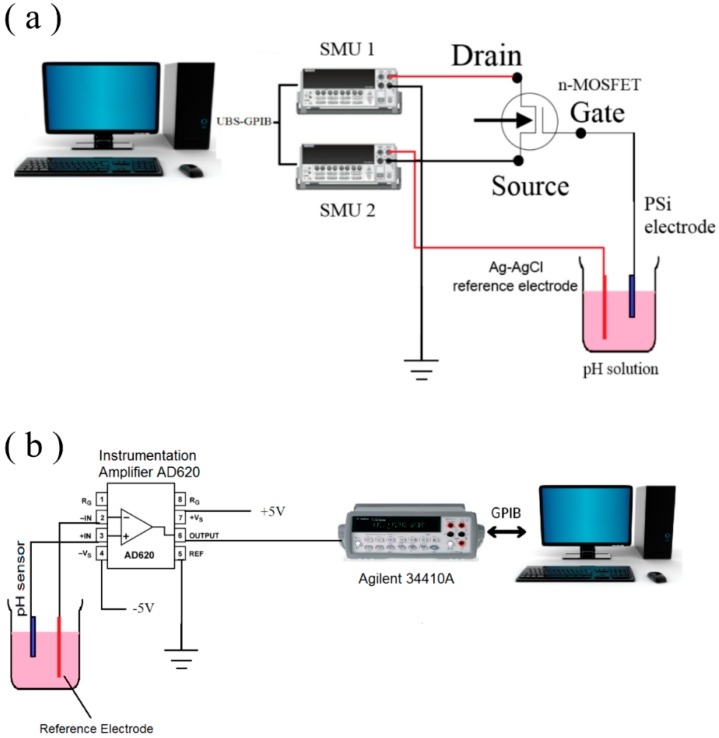
The pH-EGFET measurement setup. The measurement configuration of (**a**) I-V characteristics and (**b**) the hysteresis effect.

Two Keithley 2400 source measure units (SMUs) (Keithley Instruments, Inc., Cleveland, OH, USA) were used to analyze the membrane. The units were connected to a personal computer (PC) via a GPIB–USB cable, and LabTracer software (Keithley Instruments, Inc., Cleveland, OH, USA) was used to initiate the measurements and save the data for further analysis. The measurement system in this study is shown in Figure 3a. The electrode containing PSi was connected to the gate terminal of a commercial MOSFET (CD4007UB-Texas Instruments) and dipped into a buffer solution. To provide a stable reference voltage to the sensing element, a commercial Ag-AgCl reference electrode was dipped into the same buffer solution and kept at room temperature (25 °C ± 1 °C) for two mins before the measurements. One of the 2400 SMUs was used to apply the drain-source voltage (V_DS_) to the source and drain the terminals of the CD4007UB device and measure the drain-source output current (I_DS_), whereby the second 2400 SMU was used to apply the reference voltage (V_REF_) to the reference electrode. The distance between the reference electrode and the PSi sensing electrode was fixed at 1.7 cm.

The hysteresis effect of the prepared PSi sensing membrane was measured using an instrumentation amplifier AD620 [40]. Figure 3b depicts the schematic of the measurement configuration for the hysteresis effect. The membrane was connected to Terminal 3 of the device, whereas the reference electrode was connected to Terminal 2. The operating voltage at Terminals 4 and 7 was +5 V and −5 V, respectively. The sensor was kept for 12 hours in a buffer solution of pH 7 so the membrane surface stabilized prior to the measurement. The multimeter Agilent 34410A was used to readout the data, and it was connected to the output terminal (Terminal 6) of the AD620. In addition, the Agilent 34410A was connected to a PC through a USB interface, and the data were stored in the PC. For this test, we immersed the membrane in alternating cycles of pH buffer solutions starting from pH 7 → pH 4 → pH 7 → pH 10 → pH 7 for 60 s for each pH buffer solution. The standard phosphate buffer solutions with (pH = 2 to 12) were purchased from HANNA instruments. All the measurements were conducted in a black box, and the temperature was controlled at 25 °C ± 1 °C.

The surface morphologies of the prepared PSi were investigated using field-emission scanning electron microscopy (FE-SEM), which was conducted on a LEO SUPRA 55VP (Carl Zeiss) using energy-dispersive X-ray spectroscopy (EDX; Oxford Inca) to determine the elemental compositions.

## 3. Results and Discussion

### 3.1. The PSi Surface Morphology

**Figure 4:**
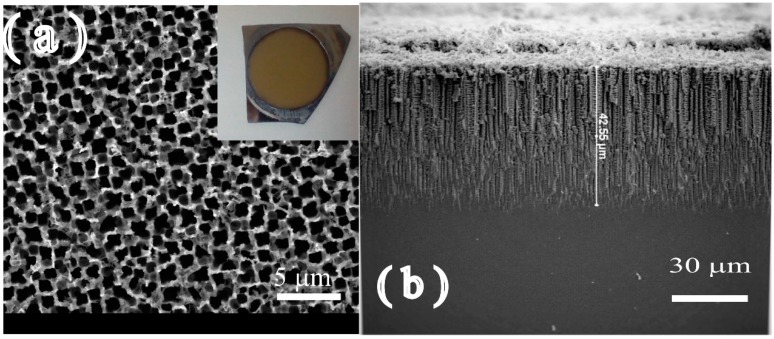
FE-SEM image of the surface: (**a**) inset depicts the optical image of the PSi surface and (**b**) cross section of the prepared PSi.

The surface morphology of the prepared PSi at the current density of 20 mA/cm^2^ for 15 mins under illumination of 100 W is depicted in Figure 4.

The FE-SEM image of the top view of the prepared PSi sample can be seen in Figure 4a, and it is clear that the pores are homogenous in their distribution. An average pore size of 500 nm to 750 nm was observed in the prepared sample. The optical image (inset in Figure 4a) illustrates the homogeneous surface of the prepared PSi sample. The figure also depicts a part of the cross section of the prepared PSi with a depth of approximately 42 μm (Figure 4b).

The EDX scan of the surface shows the presence of three elements: carbon, oxygen, and silicon. The carbon probably originated from the etching process. The oxygen is probably in the form of SiO_x_ and originated from the replacement of the Si/H terminal bonds to more stable Si/O bonds and can be presented as [14]:

(3) $$SiH_{4}+2H_{2}O\to SiO_{2}+4H_{2}$$

### 3.2. The PSi Performance as a pH Sensor

The EGFET turn-on voltage (threshold voltage; V_T_) varies with the surface potential between the sensing film and the solution. The V_T_ can be expressed as [22,23]:

(4) $$V_{T(EGFET)}=V_{T(\mathrm{MOSFET})}-\frac{\Phi_{M}}{q}{+E}_{REF}+\chi^{Sol}-\phi$$

where V_T(MOSFET)_ is the threshold voltage of the MOSFET, $\frac{\Phi_{M}}{q}$ is the metal gate work function, E_REF_ is the potential of the reference electrode, $\chi^{Sol}$ is the surface dipole potential of the buffer solution, and ϕ is the surface potential at the electrolyte/sensing film interface.

The relationship between I_DS_ and V_REF_ in the linear region of the EGFET can be expressed as [22,23];

(5) $$I_{DS}=K_{n}[2(V_{REF}-V_{T})V_{DS}-V_{DS}^{2}]$$

where *K_n_* is the conduction parameter [22].

**Figure 5:**
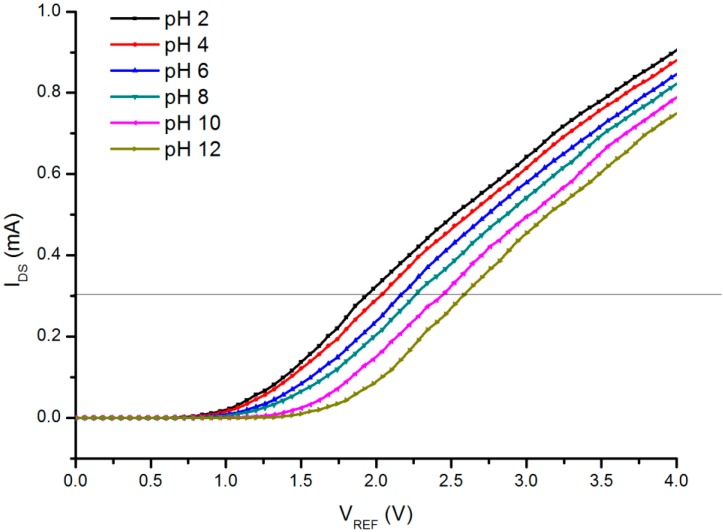
The *I_DS_* – *V_REF_* for the prepared PSi EGFET in the linear region for different pH buffer solutions (pH = 2 to 12). The *V_DS_* was kept constant at 300 mV.

The results of I_DS_ and V_REF_ are depicted in Figure 5. The V_DS_ was kept constant at 300 mV (the linear region of the MOSFET).

As seen in the figure, as the pH value increases (the H^+^ ion content decreases), the V_T_ of the EGFET is shifted to higher values (shifted from the left toward the right). From the graph, the pH voltage sensitivity and linearity can be calculated using the below formula:

(6) $$pH\;voltage\;senstivity=\frac{\Delta V_{T}}{\Delta pH}$$

**Figure 6:**
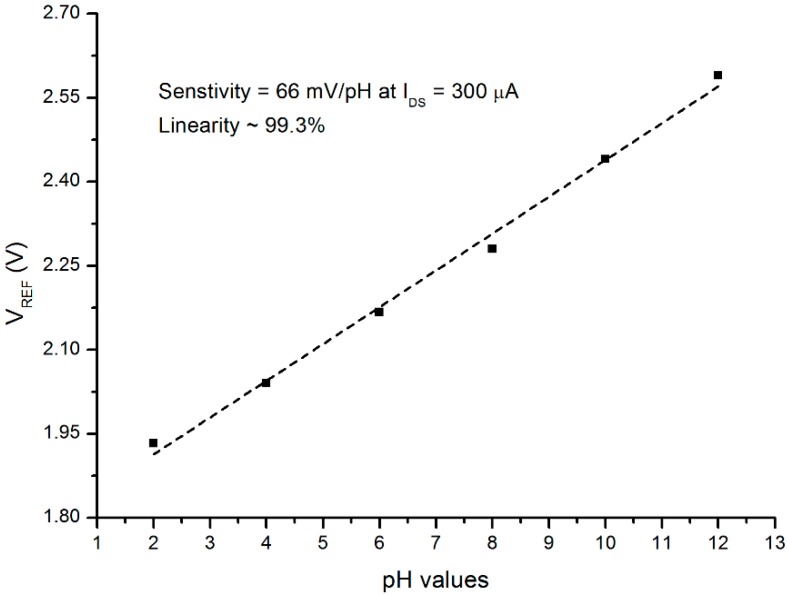
Sensitivity and linearity of prepared PSi EGFET *vs*. different pH buffer solutions (pH = 2 to 12). Black dots are measured data, and dashed line shows linear fit. I_DS_ was kept constant at 300 µA.

In Figure 6, the relationship of the V_T_ and pH values in the range of 2 to 12 is revealed. From this graph, at a constant current of I_DS_ = 300 μA, the sensitivity was found to be approximately 66 mV/pH with a linearity of 99.3%.

Three samples were measured using the same process and circumstances; the results were 63.7, 66, and 68.1 mV/pH with an average value of 65.9 mV/pH with a standard deviation of ±2. It was previously reported that the theoretical predictions from the Nernstian law indicate an upper limit for the EGFET value at about 59 mV/pH [23]. However, several reports revealed super Nernstian values can be achieved. The highest values from the reported articles were 62.87 mV/pH for PdO [23], 61.44 and 62.0 mV/pH for TiO_2_ [22,41], and 71.4 mV/pH for Pd-PdO [42]. Moreover, it was reported that a mixture of vanadium with 5% tungsten oxide achieved a sensitivity value of 68 mV/pH [43].

According to Zehfroosh *et al.* [21] and Yao *et al.* [22], the porous nature of the membrane surface will enhance the effective adsorption surface, which will result in the enhancement of the pH sensitivity of the sensor due to the increase of the charge accumulation, as more charges are accumulated, the current rises.

For this, Zehfroosh *et al.* suggested a factor be added to Equation (5). The factor is related to the concentration of the hydrogen ions and the porosity of the membrane. In the view of the results obtained in this study, we believe that the main reaction is occurred between the ions (H^+^ and OH^−^) and the available sites on the surface of the PSi. Furthermore, the porosity of the prepared PSi and the accumulation of the charge play significant roles in the enhancement of the pH voltage sensitivity as more sites will be available for the ions to react with. On another word, the higher surface-to-volume ratio with larger effective surface area of the PSi are probably the main reason of the enhancement of the pH voltage sensitivity [44].

According to the Nernst equation, the error is 0.2 mV/pH per degree (°K) at sensitivity of 59 mV/pH at 293 °K [23,42]. In this experiment, the temperature was controlled at 25 °C ± 1 °C (297 °K to 299 °K). The error at 2 °K will be 0.4 mV, which is within the experimental error, and the temperature effect on the sensor output is insignificant.

**Figure 7:**
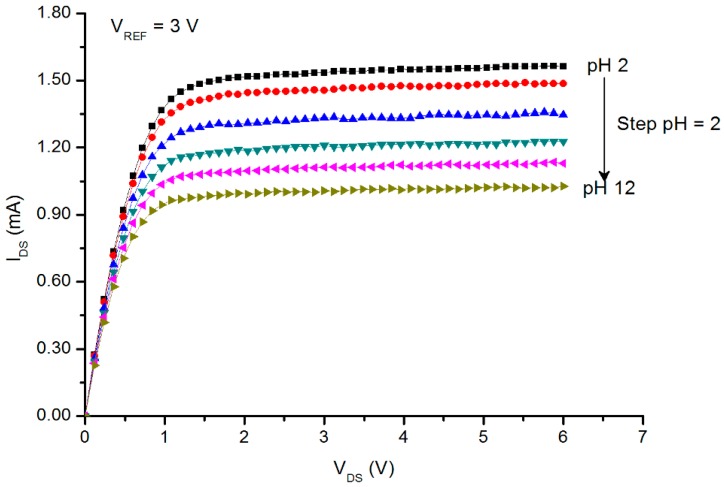
Plot of the I_DS_ *vs.* the V_DS_ of the prepared PSi pH sensor. V_REF_ was kept constant at 3 V. Buffer solutions were pH = 2–12.

The I_DS_ – V_DS_ curves (also known as the saturated region) are depicted in Figure 7 (the V_REF_ was kept constant at 3 V). Here, the I_DS_ values were shifted downward as the pH values increased. This is due to the decrease in the H^+^ ion concentrations and the increase in the OH^−^ ions, as the buffer solution altered from acidic to basic. Saturation sensitivity and linearity can be derived from Figure 7. The sensitivity of the sensor from the saturated region can be calculated as:

(7) $$\text{pH current senstivity}=\frac{\Delta \sqrt{I_{DS}}}{\Delta pH}$$

**Figure 8:**
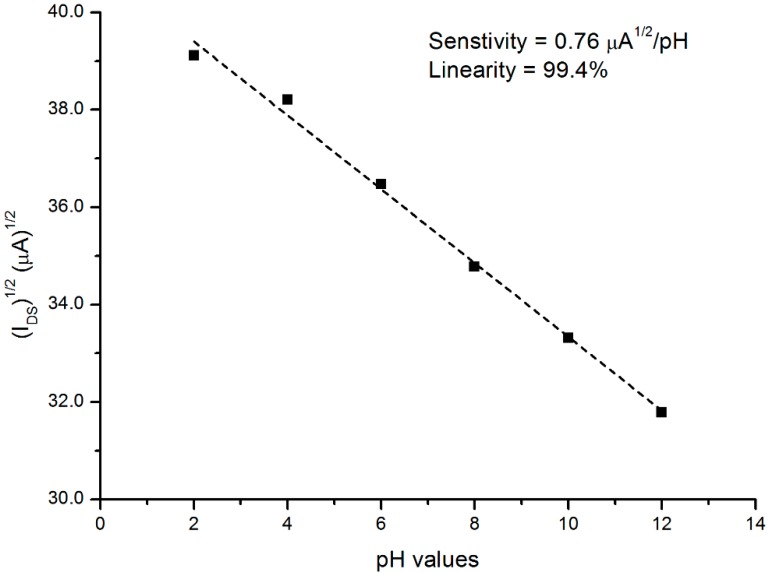
Plot of the $\surd I_{DS}$ as a function of pH for the prepared PSi pH sensor. *V_DS_* and *V_REF_* = 3 V. Black squares are measured data, and dashed line shows linear fit.

From Figure 8, the pH sensitivity was 0.76 μA^1/2^/pH with a linearity of 99.4%, and the values of I_DS_ were chosen at a constant V_DS_ = 3 V. Table 1 reveals the results of the pH current sensitivity published by several authors.

Table 1 reveals the pH current sensitivity obtained by several research groups, based on different materials, compared with the results of this study.

**Figure 9:**
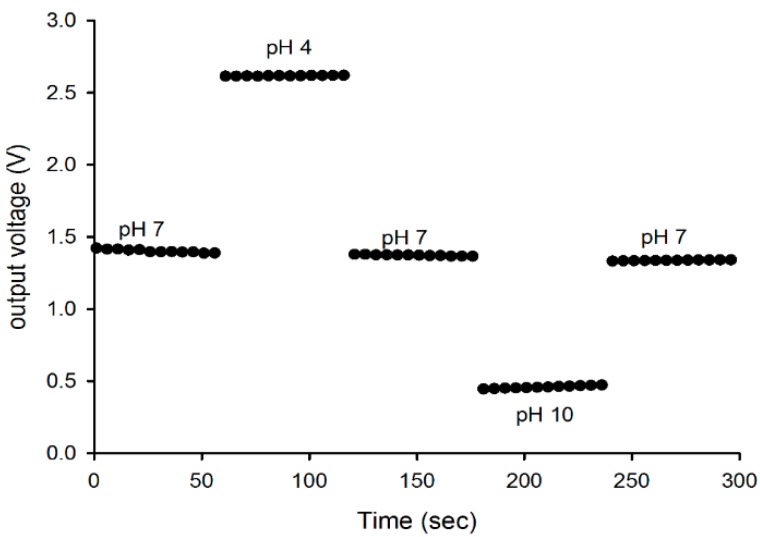
Hysteresis characteristics for prepared PSi membrane EGFET pH sensor.

Figure 9 depicts the hysteresis effect, and it was noticed that the hysteresis of the membrane was 8.2 mV for the pH 7 → pH 4 → pH 7 loop, and it was increased to 10.5 mV for the pH 7 → pH 10 → pH 7 loop. The difference in the hysteresis value between the low pH loop and the high pH loop is due to the difference in the diffusion rates for H^+^ and OH^−^ ions in the surface-sites underneath the membrane surface (buried sites) [22].

The hysteresis is known to be related to the chemical interaction between the ions (H^+^ and OH^−^) in the electrolyte [46] and the slow reacting buried sites of the membrane surface and/or the surface defects of the membrane. Since the diffusion of H^+^ ions into the buried sites of the sensing film is more rapid than that of the OH^−^ ions, the hysteresis is more significant in an alkaline solution [22,23]. The results obtained in this study are slightly higher than that of the previous studies using metal–oxide–semiconductor membranes [23,39].

The performance of the prepared PSi in this study reveals a promising behavior in the field of pH measurements based on EGFET platform with a stable performance within the maximum and minimum pH range (2–12). According to Zhou *et al.* [47] and Fulati [44] ZnO nanostructure show a degradability at different pH values. Furthermore, several studies showed the disintegration of metal oxide semiconductors such as tin oxide [29] and tungsten trioxide [48] for pH sensing applications. On the other hand, SiO_2_ (on the surface of PSi) was reported to undergo a low dissolution rate compared with the above oxides [46,47]. This might be the reason for the stable performance. It is worth noting that SiO_2_ has been used frequently in ISFET for pH and other bio analytic detections [49]. The only drawback of using SiO_2_ as a membrane was the hysteresis effect with the alkaline aqueous solutions [46].

## 4. Conclusions

In summary, we report for the first time on the usage of PSi as a membrane for hydrogen ion sensing in a buffer solution with different pH values based on EGFET platform. The PSi membrane demonstrates a high value of sensitivity at 66 mV/pH in the pH value range between 2 and 12. In view of the results obtained in this study, we believe that the porosity of the surface increases the accumulation of the charges that result in an increase of the sensitivity. The PSi membrane shows reasonably values of hysteresis between the pH values.

## Figures and Tables

**Table 1:** The pH current sensitivity for various materials.

Sensing Membrane	Platform	Current Sensitivity μA^1/2^/pH	Reference
PbO thin film	EGFET	1.08	[23]
V_2_O_5_/WO_3_ thin film	EGFET	1.36	[43]
ZnO/Si nanowire	EGFET	0.73	[45]
ZnO thin film	EGFET	0.54	[45]
PSi	EGFET	0.76	this study

## References

[1] Harraz F.A. (2014). Porous silicon chemical sensors and biosensors: A review. Sens. Actuators B Chem..

[2] Palestino G., Legros R., Agarwal V., Pérez E., Gergely C. (2008). Functionalization of nanostructured porous silicon microcavities for glucose oxidase detection. Sens. Actuators B Chem..

[3] Dhanekar S., Islam S.S., Islam T., Shukla A.K. (2010). Organic vapour sensing by porous silicon: Influence of molecular kinetics in selectivity studies. Phys. E Low-Dimens. Syst. Nanostruct..

[4] Dhanekar S., Jain S. (2013). Porous silicon biosensor: Current status. Biosens. Bioelectron..

[5] RoyChaudhuri C. (2015). A review on porous silicon based electrochemical biosensors: Beyond surface area enhancement factor. Sens. Actuators B Chem..

[6] López-García J., Martín-Palma R.J., Manso M., Martínez-Duart J.M. (2007). Porous silicon based structures for the electrical biosensing of glucose. Sens. Actuators B Chem..

[7] Koshida N., Koyama H. (1992). Visible electroluminescence from porous silicon. Appl. Phys. Lett..

[8] Fauchet P.M., von Behren J., Hirschman K.D., Tsybeskov L., Duttagupta S.P. (1998). Porous Silicon Physics and Device Applications: A Status Report. Phys. Status Solidi.

[9] Canham L.T., Cox T.I., Loni A., Simons A.J. (1996). Progress towards silicon optoelectronics using porous silicon technology. Appl. Surf. Sci..

[10] Ünal B., Parbukov A.N., Bayliss S.C. (2001). Photovoltaic properties of a novel stain etched porous silicon and its application in photosensitive devices. Opt. Mater..

[11] Balagurov L.A., Bayliss S.C., Yarkin D.G., Andrushin S.Y., Kasatochkin V.S., Orlov A.F., Petrova E.A. (2003). Low noise photosensitive device structures based on porous silicon. Solid-State Electron..

[12] Hadjersi T., Gabouze N. (2008). Photodetectors based on porous silicon produced by Ag-assisted electroless etching. Opt. Mater..

[13] Balucani M., Bondarenko V., Klusko A., Ferrari A. (2005). Recent progress in integrated waveguides based on oxidized porous silicon. Opt. Mater..

[14] Liyanage C.N., Blackwood D.J. (2014). Functionalization of a porous silicon impedance sensor. Thin Solid Films.

[15] Massera E., Nasti I., Quercia L., Rea I., Di Francia G. (2004). Improvement of stability and recovery time in porous-silicon-based NO_2_ sensor. Sens. Actuators B Chem..

[16] Mahmoudi B., Gabouze N., Haddadi M., Mahmoudi B., Cheraga H., Beldjilali K., Dahmane D. (2007). The effect of annealing on the sensing properties of porous silicon gas sensor: Use of screen-printed contacts. Sens. Actuators B Chem..

[17] Kanungo J., Saha H., Basu S. (2009). Room temperature metal–insulator–semiconductor (MIS) hydrogen sensors based on chemically surface modified porous silicon. Sens. Actuators B Chem..

[18] Anglin E.J., Cheng L., Freeman W.R., Sailor M.J. (2008). Porous silicon in drug delivery devices and materials. Adv. Drug Deliv. Rev..

[19] Chen C.-C., Chen H.-I., Liu H.-Y., Chou P.-C., Liou J.-K., Liu W.-C. (2015). On a GaN-based ion sensitive field-effect transistor (ISFET) with a hydrogen peroxide surface treatment. Sens. Actuators B Chem..

[20] Xu F., Yan G., Wang Z., Jiang P. (2015). Continuous accurate pH measurements of human GI tract using a digital pH-ISFET sensor inside a wireless capsule. Measurement.

[21] Zehfroosh N., Shahmohammadi M., Mohajerzadeh S. (2010). High-Sensitivity Ion-Selective Field-Effect Transistors Using Nanoporous Silicon. IEEE Electron Device Lett..

[22] Yao P.-C., Chiang J.-L., Lee M.-C. (2014). Application of sol–gel TiO_2_ film for an extended-gate H+ ion-sensitive field-effect transistor. Solid State Sci..

[23] Das A., Ko D.H., Chen C.-H., Chang L.-B., Lai C.-S., Chu F.-C., Chow L., Lin R.-M. (2014). Highly sensitive palladium oxide thin film extended gate FETs as pH sensor. Sens. Actuators B Chem..

[24] Batista P.D., Mulato M. (2005). ZnO extended-gate field-effect transistors as pH sensors. Appl. Phys. Lett..

[25] Chiu Y.-S., Tseng C.-Y., Lee C.-T. (2012). Nanostructured EGFET pH Sensors With Surface-Passivated ZnO Thin-Film and Nanorod Array. IEEE Sens. J..

[26] Liao Y.-H., Chou J.-C. (2009). Preparation and characterization of the titanium dioxide thin films used for pH electrode and procaine drug sensor by sol–gel method. Mater. Chem. Phys..

[27] Sardarinejad A., Maurya D., Alameh K. (2015). The pH Sensing Properties of RF Sputtered RuO_2_ Thin-Film Prepared Using Different Ar/O_2_ Flow Ratio. Materials.

[28] Batista P., Mulato M. (2010). Polycrystalline fluorine-doped tin oxide as sensoring thin film in EGFET pH sensor. J. Mater. Sci..

[29] Chou J.-C., Kwan P.K., Chen Z.-J. (2003). SnO_2_ Separative Structure Extended Gate H^+^ Ion Sensitive Field Effect Transistor by the Sol–Gel Technology and the Readout Circuit Developed by Source Follower. Jpn. J. Appl. Phys..

[30] Reddy R.R.K., Chadha A., Bhattacharya E. (2001). Porous silicon based potentiometric triglyceride biosensor. Biosens. Bioelectron..

[31] Reddy R.R.K., Basu I., Bhattacharya E., Chadha A. (2003). Estimation of triglycerides by a porous silicon based potentiometric biosensor. Curr. Appl. Phys..

[32] Schöning M., Simonis A., Ruge C., Ecken H., Müller-Veggian M., Lüth H. (2002). A (Bio-) Chemical Field-Effect Sensor with Macroporous Si as Substrate Material and a SiO_2_/LPCVD-Si_3_N_4_ Double Layer as pH Transducer. Sensors.

[33] Yates D.E., Levine S., Healy T.W. (1974). Site-binding model of the electrical double layer at the oxide/water interface. J. Chem. Soc. Faraday Trans. 1 Phys. Chem. Condens. Phases.

[34] Tae-Eon B., Hyun-June J., Se-Won L., Won-Ju C. (2013). Enhanced Sensing Properties by Dual-Gate Ion-Sensitive Field-Effect Transistor Using the Solution-Processed Al_2_O_3_ Sensing Membranes. Jpn. J. Appl. Phys..

[35] Oldham K.B. (2008). A Gouy–Chapman–Stern model of the double layer at a (metal)/(ionic liquid) interface. J. Electroanal. Chem..

[36] Pan T.-M., Huang M.-D., Lin C.-W., Wu M.-H. (2010). Development of high-κ HoTiO_3_ sensing membrane for pH detection and glucose biosensing. Sens. Actuators B Chem..

[37] Parizi K.B., Yeh A.J., Poon A.S.Y., Wong H.S.P. Exceeding Nernst limit (59 mV/pH): CMOS-based pH sensor for autonomous applications, Electron Devices Meeting (IEDM). Proceedings of the 2012 IEEE International.

[38] Spijkman M., Smits E.C.P., Cillessen J.F.M., Biscarini F., Blom P.W.M., de Leeuw D.M. (2011). Beyond the Nernst-limit with dual-gate ZnO ion-sensitive field-effect transistors. Appl. Phys. Lett..

[39] Mahmoud N., Hassan Z., Abd H.R. (2012). Design of Metal-Semiconductor-Metal Photodetector: Porous Silicon Photodetector.

[40] Yin L.-T., Chou J.-C., Chung W.-Y., Sun T.-P., Hsiung S.-K. (2000). Separate structure extended gate H^+^ ion sensitive field effect transistor on a glass substrate. Sens. Actuators B Chem..

[41] Huang Y.-C., Tai F.-S., Wang S.-J. (2014). Preparation of TiO_2_ nanowire arrays through hydrothermal growth method and their pH sensing characteristics. Jpn. J. Appl. Phys..

[42] Liu C.-C., Bocchicchio B.C., Overmyer P.A., Neuman M.R. (1980). A Palladium-Palladium Oxide Miniature pH Electrode. Science.

[43] Guidelli E.J., Guerra E.M., Mulato M. (2012). V_2_O_5_/WO_3_ Mixed Oxide Films as pH-EGFET Sensor: Sequential Re-Usage and Fabrication Volume Analysis. ECS J. Solid State Sci. Technol..

[44] Fulati A., Usman Ali S.M., Riaz M., Amin G., Nur O., Willander M. (2009). Miniaturized pH Sensors Based on Zinc Oxide Nanotubes/Nanorods. Sensors.

[45] Li H.-H., Yang C.-E., Kei C.-C., Su C.-Y., Dai W.-S., Tseng J.-K., Yang P.-Y., Chou J.-C., Cheng H.-C. (2013). Coaxial-structured ZnO/silicon nanowires extended-gate field-effect transistor as pH sensor. Thin Solid Films.

[46] Luc B., Bergveld P. (1984). The Role of Buried OH^−^ Sites in the Response Mechanism of Inorganic-Gate pH-Sensitive ISFETs. Sens. Actuators.

[47] Zhou J., Xu N., Wang Z.L. (2006). Dissolving Behavior and Stability of ZnO Wires in Biofluids: A Study on Biodegradability and Biocompatibility of ZnO Nanostructures. Adv. Mater..

[48] Chou J.C., Chiang J.L. (2000). Study on the amorphous tungsten trioxide ion-sensitive field effect transistor. Sens. Actuators B Chem..

[49] Luo X., Xu J., Zhao W., Chen H. (2004). Glucose biosensor based on ENFET doped with SiO_2_ nanoparticles. Sens. Actuators B Chem..

